# Inspiratory muscle training in stroke patients with congestive heart failure: A CONSORT-compliant prospective randomized single-blind controlled trial: Erratum

**DOI:** 10.1097/MD.0000000000006212

**Published:** 2017-02-17

In the article “Inspiratory muscle training in stroke patients with congestive heart failure: A CONSORT-compliant prospective randomized single-blind controlled trial”,^[1]^ which appeared in Volume 95, Issue 37 of *Medicine*, a footnote should have been included noting that Mei-Yun Liaw is the co-first author of the article.
